# Recovery of Iodine in the Gaseous Phase Using the Silicone Hollow Fiber Membrane Module

**DOI:** 10.3390/membranes15010027

**Published:** 2025-01-13

**Authors:** Yoshio Yamabe, Naotake Takahashi, Jun Sawai, Tamotsu Minami, Mikio Kikuchi, Toshimitsu Ishii

**Affiliations:** 1 Department of Applied Chemistry, Faculty of Engineering, Kanagawa Institute of Technology, 1030 Shimo-Ogino, Atsugi 243-0292, Japan; 2 Department of Nutrition and Life Science, Faculty of Health and Medical Sciences, Kanagawa Institute of Technology, 1030 Shimo-Ogino, Atsugi 243-0292, Japan; 3 Faculty of Applied Bioscience, Kanagawa Institute of Technology, 1030 Shimo-Ogino, Atsugi 243-0292, Japan; 4 Godo Shigen Co., Ltd., 1545-1 Nanaido, Chosei-mura, Chiba 299-4333, Japan; t.ishii@godoshigen.co.jp

**Keywords:** membrane separation, permeation and chemical desorption method, polydimethylsiloxane, silicone hollow fiber membrane, iodine

## Abstract

Iodine, being an important resource, must be recovered and reused. Iodine is not only attracted to the hydrophobic silicone membrane but also easily vaporized. In this study, we explored the use of five types of silicone hollow fiber membrane modules (SFMMs) for separating iodine in the gaseous phase. In the SFMM, iodine gas and the recovery solution (sodium sulfite and sodium carbonate at a concentration of 10 mM each) were flowed outside and inside the silicone hollow fiber, respectively, in a co-current-flow manner. At an iodine gas flow rate of 0.2 L/min (8.4 × 10^−3^ mmol-I_2_/L), the capture efficiency of iodine into the SFMM was approximately 100% for all five SFMMs. With increasing feed gas flow rates, the capture efficiency of iodine decreased, reducing to approximately 50% at 0.8 L/min. However, the recovery efficiency of iodine in the recovery solution was 60–30% at 0.2–0.8 L/min. This decrease in capture efficiency with increasing flow rates was because iodine could not spread and diffuse successfully in the SFMM, resulting in a low recovery efficiency of iodine. Thus, we next improved the structure of the SFMM by placing a perforated pipe in the center of the module. The perforated pipe effectively directs the iodine feed gas from the holes in the pipe to the hollow fiber membrane bundle wrapped around the pipe. With the improved SFMM, the capture efficiency markedly increased to approximately 100% in the range of the flow rates tested in our experiments. The recovery efficiency also increased to ≥70%. These data illustrate the potential application of the improved SFMM for recovering iodine in the gaseous phase.

## 1. Introduction

Iodine is used in numerous applications, such as X-ray contrast media [1,2], disinfectants [3,4], photosensitizers, catalysts, stabilizers, polarizing films on liquid crystal displays, and reaction intermediates [5,6], because of its high reactivity. Potassium iodide is also often added to table salt and feed to prevent iodine deficiency [7].

The blowing-out method is generally used for processing large amounts of iodine or brine at high temperatures. The first stage in this method is chlorine oxidation to convert iodide into iodine. The iodine is then removed from the brine as vapor. The iodine vapor is recovered using absorption [5], solvent extraction, activated carbon adsorption [8], or ion exchange [9]. Although this processing method is suitable for both large and small plants, solvent extraction and activated carbon and ion exchange treatments also use a large amount of organic solvent or oxidant agents during the recovery of iodine. Hence, from the viewpoint of cost and environmental impact, these processing methods are certainly not ideal.

Iodine and iodine compounds, in some cases, do not remain in the products but are discharged after their use in reactions. If this iodine is released into the environment, it will harm the environment; this situation occurs in the case of X-ray contrast media and disinfectants [10]. The discharge of iodine into the environment also indicates the loss of a precious resource.

The membrane separation process is widely used in industries because of its numerous advantages, such as low energy consumption, low use of space, and simple process design compared with other processes [11]. Studies have reported on the solubility and diffusivity of iodine into silicone [12] and the applications of the membrane separation of iodine using gas membranes, such as Gore-Tex^™^ (W. L. Gore & Associates, Inc., Newark, DE, USA) [13]. We had proposed the permeation and chemical desorption (PCD) method as an alternative for recovering iodine [14]. A target substance can be separated and recovered from an aqueous solution by altering the characteristics of the substance from high affinity toward the membrane to poor affinity through a chemical reaction, such as neutralization or oxidation–reduction. This method provided successful results on the separation of phenols and anilines by neutralization [15,16,17,18,19].

In a previous report [14], by placing an acidic feed solution containing iodine and an alkaline recovery solution on either side of a silicone flat membrane, the iodine in the feed solution was successfully separated and recovered in the recovery solution. The dissociation of iodine in aqueous solution is expressed by Equation (1),

I_2_ + H_2_O ⇄ HIO + H^+^ + I^−^ (1)

and the hydrolysis constant *K*_h_ is expressed by Equation (2) [20]:

(2) $$K_{h}=\frac{\left[HIO\right]\left[H^{+}\right][I^{-}]}{\left[I_{2}\right]}\;=\;4.6\;\times \;10^{-13}\;(\mathrm{mol}/L{)}^{2}\;25\;{}^{\circ}C$$

**Figure 1:**
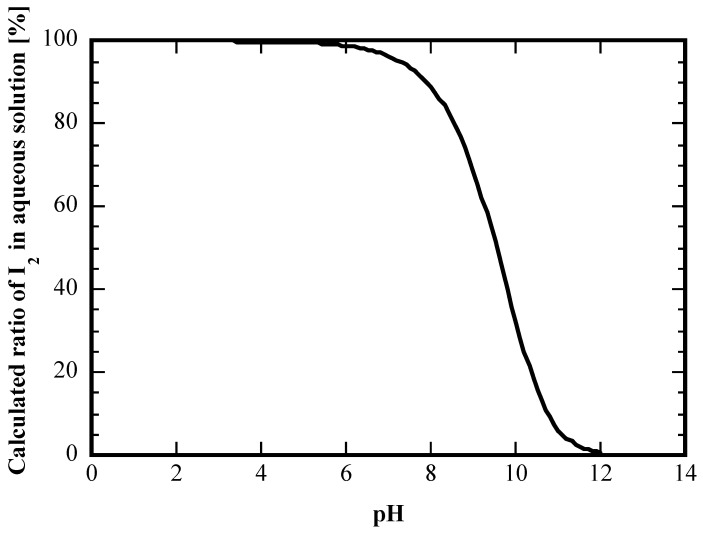
Change in the proportion of I_2_, calculated based on Equations (1) and (2), in aqueous solution with varying pH.

**Figure 2:**
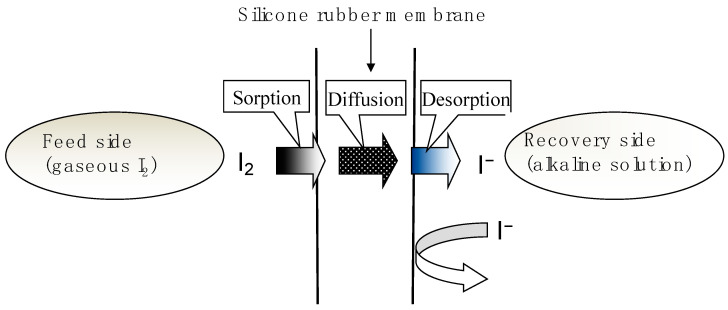
Model of permeation of gaseous I_2_ through the silicone membrane and recovery for the PCD method.

Equation (2) demonstrates the significant influence of pH on the chemical equilibrium between I_2_ and I^−^ in aqueous solution. Figure 1 presents the change in the proportion of I_2_ in aqueous solution with varying pH, which was calculated based on Equations (1) and (2). The contribution of the dissociation reactions of HIO in water (2HIO ⇄ 2HI + O_2_, 3HIO ⇄ HIO_3_ + 2HI) was omitted here for the sake of simplicity. As shown in Figure 1, in the acidic to neutral range, the iodine in the aqueous phase exists in the form of iodine molecules, while in the alkaline range, it exists in the form of iodide ions. This study employed gaseous iodine on the feed side instead of an aqueous iodine solution (Figure 2). Due to its high solubility in organic solvents, iodine is attracted to the hydrophobic silicone membrane. It can be sorbed into and permeated through the silicone membrane. When the iodine molecule diffused through the silicone membrane reaches the alkaline recovery solution, it is reduced to I^−^. The iodide ions, being negatively charged, are not absorbed by the silicone membrane. Therefore, the I^−^ in the recovery solution does not pass through the silicone membrane and return to the feed side, enabling the stable concentration and recovery of iodine from the aqueous phase (Figure 2).

Iodine is easily vaporized. The membrane separation process can improve the energy consumed and space required for recovering iodine vaporized in the blowing-out method. Moreover, using hollow fiber membrane modules makes it possible to carry out continuous operations in a small space rather than batch operations in the ion exchange processes. There are no reports of separating gaseous iodine using hollow silicon fiber membranes. This study aimed to continuously separate and recover gaseous iodine in an energy-and space-saving manner by introducing a silicone membrane hollow fiber module. The novelty of this study is that we proposed a new structure for hollow fiber membrane modules because the conventional structure of hollow fiber membrane modules could not be used to separate gaseous iodine successfully.

## 2. Materials and Methods

### 2.1. Chemicals

Iodine crystals were used without further purification. The iodine recovery solution (pH 11.1) was prepared using sodium sulfite (Na_2_SO_3_) and sodium carbonate (Na_2_CO_3_) at a concentration of 10 mM each. The concentration of the iodine recovery solution was determined in a previous study [14]. All chemicals were purchased from Kanto Kagaku Co., Ltd. (Tokyo, Japan).

### 2.2. Apparatus

**Figure 3:**
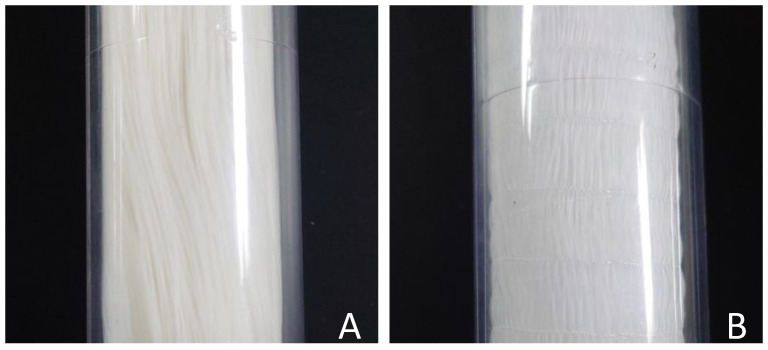
Shape of the silicone hollow fiber membrane. (**A**) straight type, (**B**) plain fabric.

**Figure 4:**
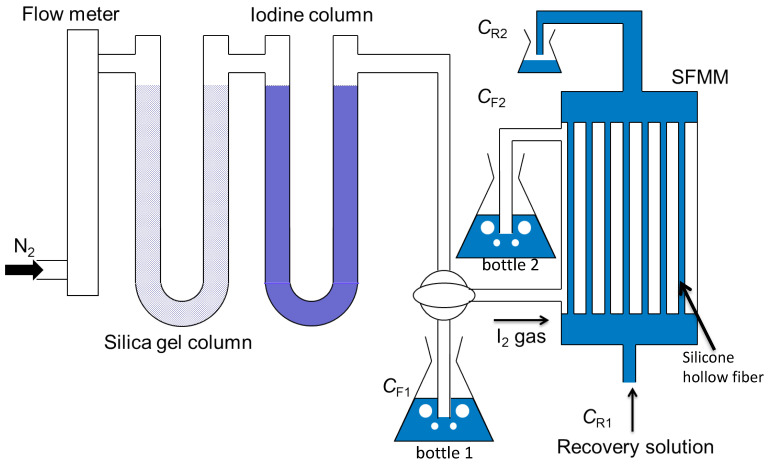
Schematic of the apparatus for recovering iodine in the gaseous phase.

The SFMM “NAGASEP™” used in this study was purchased from Nagayanagi Co., Ltd. (Tokyo, Japan). Table 1 summarizes the dimensions and configurations of the membrane modules. Two types of hollow fibers were used. In the straight type, shown in Figure 3A, each hollow fiber is separate, whereas in the plain-fabric type, shown in Figure 3B, the hollow fibers are interconnected by weft yarns and arranged at equal distances. The schematic of the apparatus used for recovering iodine in the gaseous phase is illustrated in Figure 4. Nitrogen (N_2_) was used as a carrier gas, and its flow rate was controlled using a flow meter (KOFLOC Co., Kyoto, Japan) and dehydrated through a silica gel column. Iodine was vaporized by passing the N_2_ gas through the iodine crystal column and introduced into the SFMM inlet. Initially, gaseous iodine was supplied into the hollow fibers. Gaseous iodine entered the SFMM housing from the top or bottom; however, at the distribution stage to the hollow fibers, the iodine accumulated (only the very top or bottom of the bundle of hollow fibers turned brown). As a result, it was almost impossible for the iodine to come into contact with the recovery liquid. Therefore, in the SFMM, iodine gas and the recovery solution were flowed outside and inside the silicone hollow fiber, respectively, in a co-current-flow manner (Figure 3). First, the recovery solution was flowed and filled inside the silicone hollow fiber membrane, and then the start point of the experiment (*t* = 0) was determined by the moment the I_2_ gas passed through the inlet of the hollow fiber in the modules. The experimental temperature was controlled at 25 °C ± 2 °C.

The iodine in the feed gas was absorbed to measure the concentration by passing the gas into bottle 1 containing the recovery solution (70 mL) for 5 min. The concentration of the residual iodine in the gas that passed through the SFMM was measured by passing the gas into bottle 2 containing the recovery solution (70 mL) for 5 min. Samples (1 mL) were extracted from each bottle, and iodide concentrations were determined as described in Section 2.3. We confirmed that 100% of the iodine in the feed gas could be recovered in this manner to calculate its concentration.

### 2.3. Determination of Iodine or Iodide Concentration

The concentration of iodine was measured by high-performance liquid chromatography (HPLC) coupled with a UV detector. To 1 mL of the sample solution, 0.1 mL of sodium sulfite solution (0.5 M) and 0.5 mL of acetic acid buffer solution (0.2 M) were added. This mixture was analyzed under the conditions shown in Table 2. All reagents were of analytical grade and purchased from Kanto Kagaku Co., Ltd.

### 2.4. Estimation of Capture and Recovery Amount of Iodide in the SFMMs

The capture efficiency is calculated using the following Equation (3). The collection efficiency here refers to the total amount of iodine absorbed by the silicon membrane, as well as the amount of iodine adsorbed and deposited on the membrane, and the amount of iodine accumulated and trapped in the SFMM, which is expressed as a ratio to the amount of iodine that entered the SFMM.

Capture efficiency [%] = (*C*_F1_−*C*_F2_)/*C*_F1_ × 100, (3)

where *C*_F1_ is the inlet concentration of the iodine feed [mmol/L], and *C*_F2_ is the outlet concentration of iodine, as shown in Figure 4.

The recovery efficiency was calculated using the following equation:

Recovery efficiency [%] = *R*_1_/*F*_1_ × 100, (4)

where *F*_1_ = *C*_F1_ × *V*_F_ and *R*_1_ = *C*_R2_ × *V*_R_. *C*_R2_ is the outlet concentration of iodine in the recovery solution. *V*_F_ and *V*_R_ are the feed gas flow rate and recovery solution flow rate [L/min], respectively.

### 2.5. Display of Data

Each experiment of SFMMs was performed in duplicate (*n* = 2). The upper and lower limits of the bars in the figure indicate the respective data, and the mean of the two points is plotted.

## 3. Results and Discussion

### 3.1. Recovery of Iodine in the Gaseous Phase

**Figure 5:**
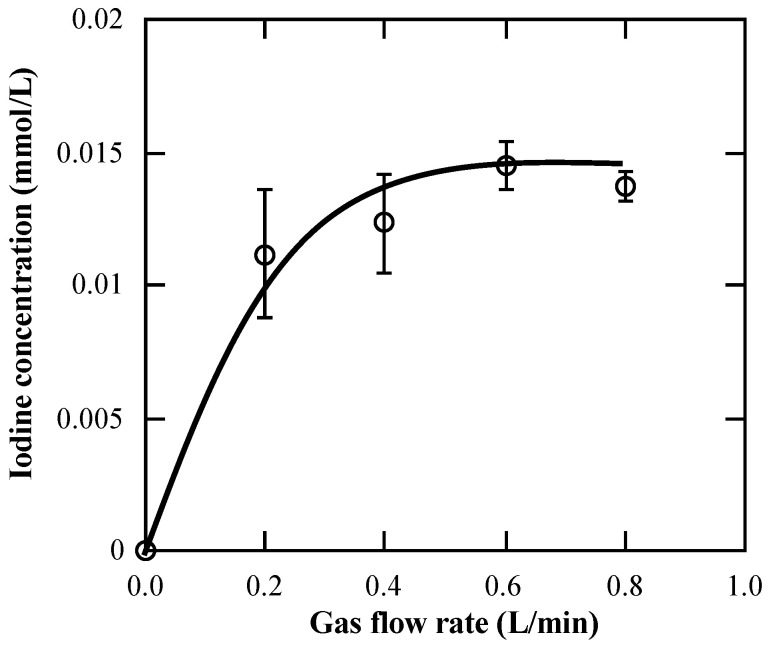
Relationship between inlet gas flow rate and iodine concentration. Data points with bars represent mean ± standard error (*n* = 10) values.

**Figure 6:**
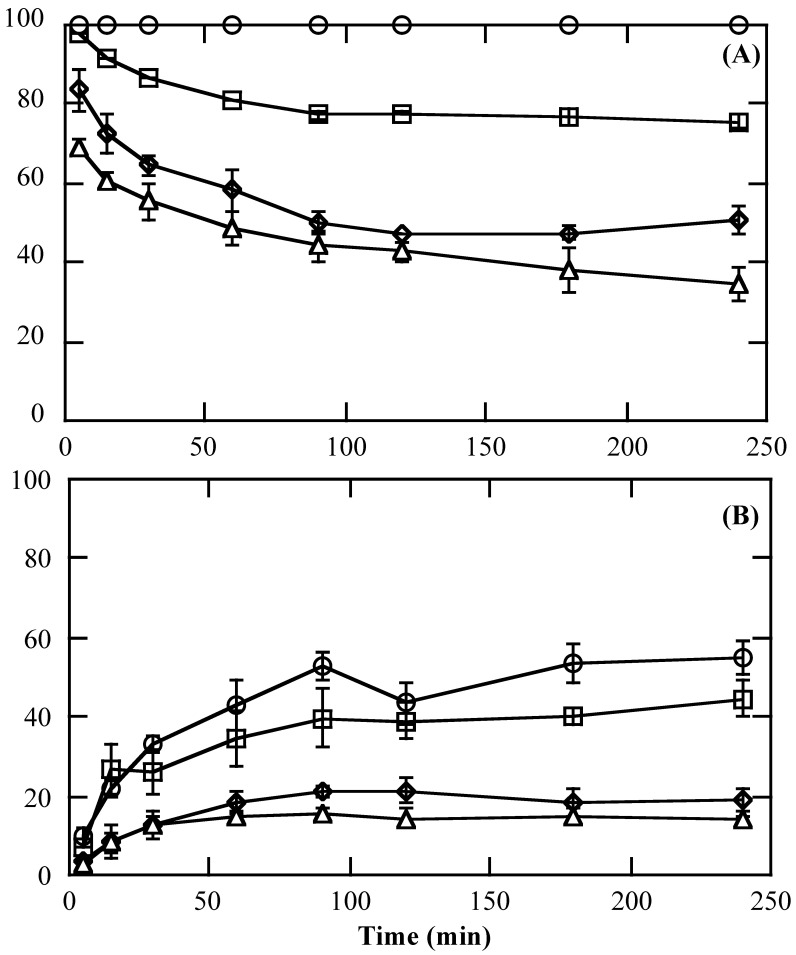
Typical tendencies of capture efficiency (**A**) and recovery efficiency (**B**) of iodine in the gaseous phase using an SFMM of M60-3000 (70S). Symbols: ○: 0.2; □: 0.4; ◊: 0.6; △: 0.8 L/min. The upper and lower limits of the bars indicate the respective data, and the mean of the two points is plotted (*n* = 2).

For the feed gas, the iodine concentration was in the range of 0.011–0.015 mol-I_2_/L and almost constant, irrespective of the nitrogen gas flow rate (0.2–0.8 L/min) (Figure 5). At a feed iodine gas flow rate of 0.2 L/min, the outlet concentration of iodine was approximately zero, indicating that almost 100% of the iodine was captured into the SFMM. Over time, the capture efficiency decreased at 0.4 L/min, and the recovery efficiency increased. The inlet and outlet concentrations of iodine reached a constant value for approximately 2 h (Figure 6).

**Figure 7:**
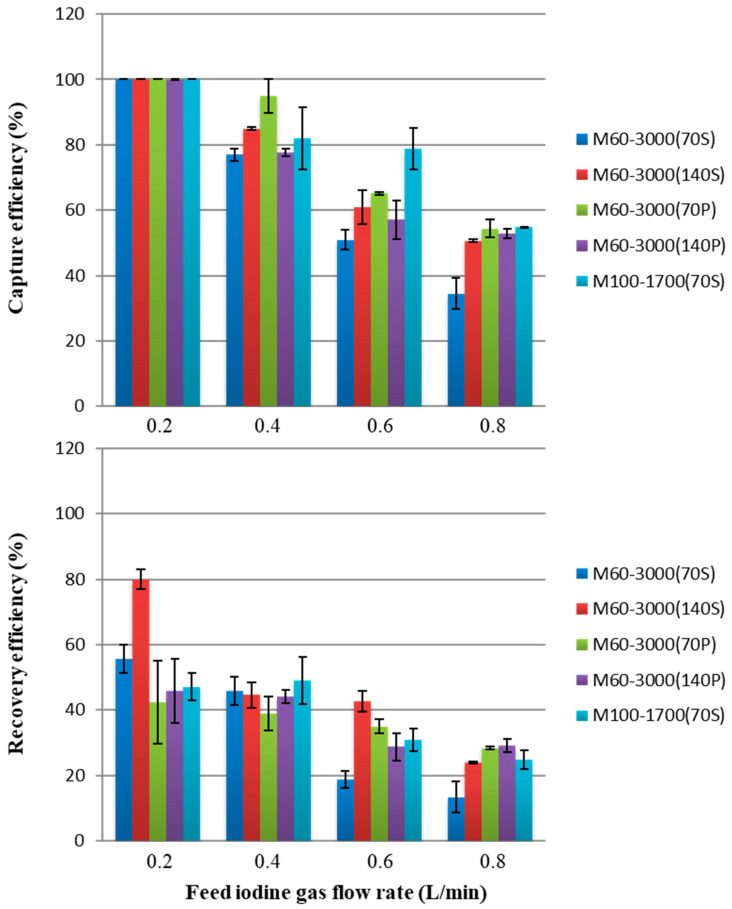
Capture efficiency and recovery efficiency of iodine in the gaseous phase using SFMMs after 2 h. The upper and lower limits of the bars indicate the respective data, and the mean of the two points is plotted (*n* = 2).

**Figure 8:**
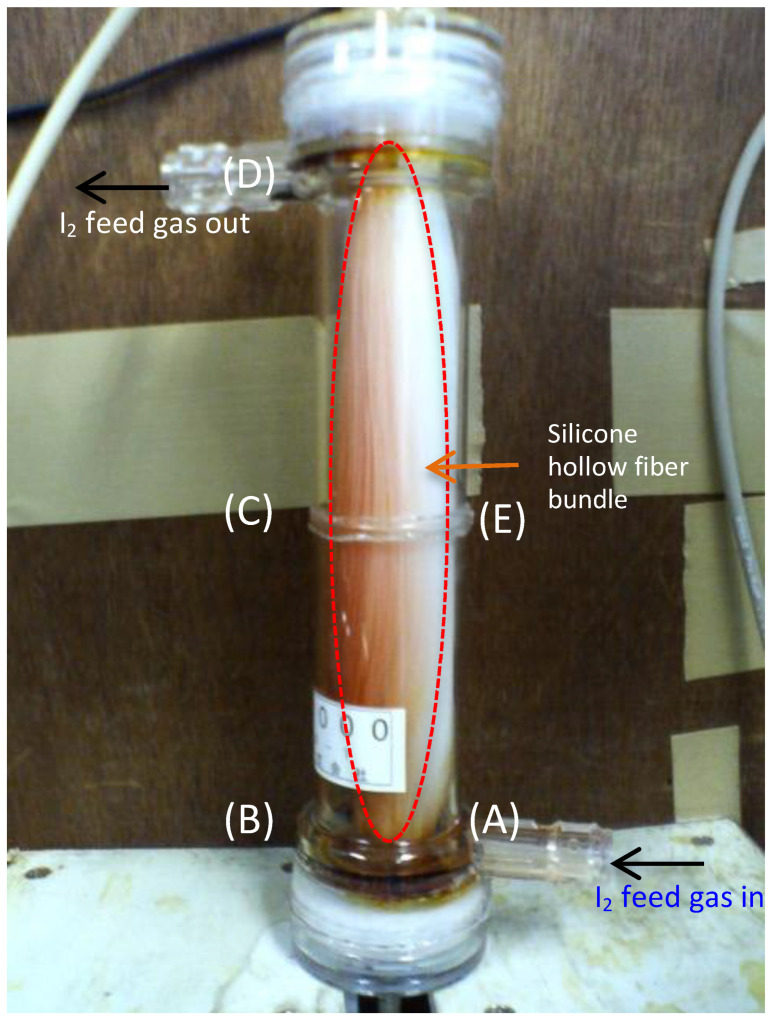
Biased sorption and one-sided flow of iodine in the SFMM.

Figure 7 summarizes the capture and recovery efficiencies after 2 h for each module. At a feed gas flow rate of 0.2 L/min, the capture efficiency was almost 100% for all types of modules, which was because the gas flow rate was low, iodine diffused and spread throughout the module, and the iodine molecules were in sufficient contact with the surface of the silicon hollow membrane. Module M60-3000 (140S) recovered more iodine than the other modules at only 0.2 L/min, but the reason was not clear. In all SFMMs, the capture efficiency generally decreased from 70% to 50% with increasing feed gas flow rates. In addition to the capture efficiency, the recovery efficiency decreased from approximately 50% to 30% (Figure 7). This phenomenon was attributed to the fact that the iodine feed gas that entered from the inlet at the bottom of the module flowed around the silicon fiber bundles but could not reach the hollow fiber interstices. Figure 8 depicts a photograph of M60-3000 (140S) when the iodine gas flowed at 0.8 L/min. According to this figure, I_2_ gas enters the module from the inlet (A) and travels around the outside of the bundle of hollow fiber membranes to reach (B). Then, the color change of the hollow fiber due to iodine (white to brown) indicates that the iodine molecules ascended inside the housing along (B) to (C) to (D) and exited the module from (D). No browning of the hollow fiber membrane due to iodine sorption was observed on the (E) side. This one-sided flow trend became more pronounced with increasing feed gas flow rates.

The recovery efficiencies were 30–60% lower than the capture efficiency at all feed gas flow rates, implying that the iodine captured in the module did not react efficiently with the recovery solution. As the iodine gas flow rate increased, the one-sided flow depicted in Figure 8 occurred, and the amount of iodine that did not contact and adsorb on the silicone membrane increased, resulting in a lower capture efficiency. The one-sided flow enabled only a small portion of the hollow fiber membrane bundle to come into contact with the iodine and adsorb it. Consequently, most of the recovered solution flowed through the hollow fiber without reacting with the iodine, and the recovery efficiency also reduced.

As shown in Figure 7, there was no significant difference in the capture and recovery efficiencies between the 70 and 140 mm modules. Moreover, the shape and number of hollow fibers exerted no significant effect on the results. The efficient contact with the silicon fibers and the iodine gas stream is essential to improve the capture and recovery efficiencies; therefore, we modified the structure of the module.

### 3.2. Improvement of the Module

**Figure 9:**
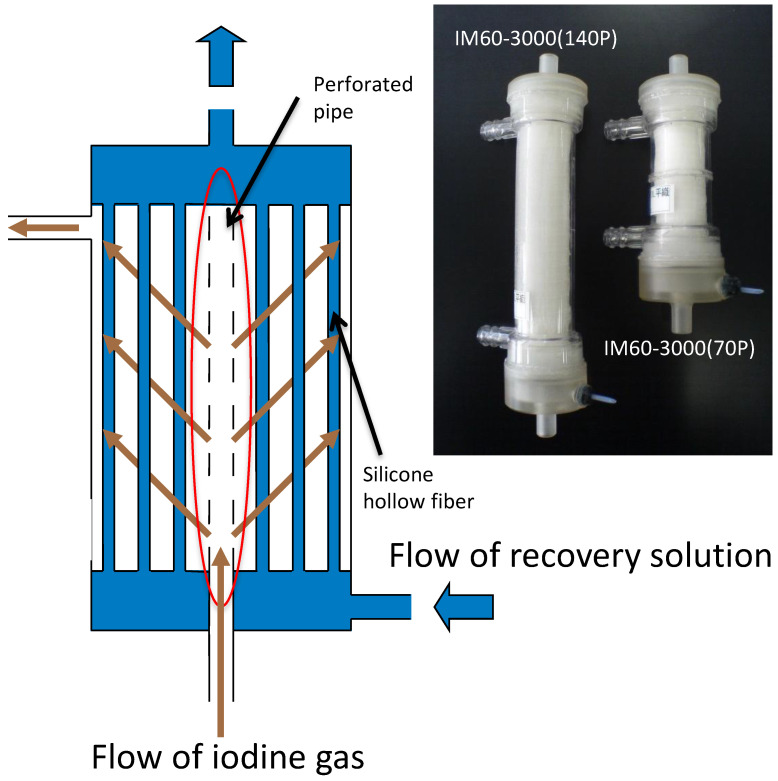
Schematic of the internal structure of the improved SFMM and flow of gas.

The structure of the modified SFMM is depicted in Figure 9. This improvement was achieved by placing a perforated pipe in the center of the module. The perforated pipe effectively directs the iodine feed gas from the holes in the pipe to the hollow fiber membrane bundle wrapped around the pipe. Iodine inevitably passes through the bundle of hollow fiber membrane on its way from the perforated pipe to the housing wall, where it efficiently contacts and is adsorbed by the silicone hollow fiber membrane. Iodine molecules adsorbed on the hollow fiber silicone membrane diffuse through the membrane and are recovered when they encounter the recovery solution. The dimensions and configuration of the module are summarized in Table 3.

**Figure 10:**
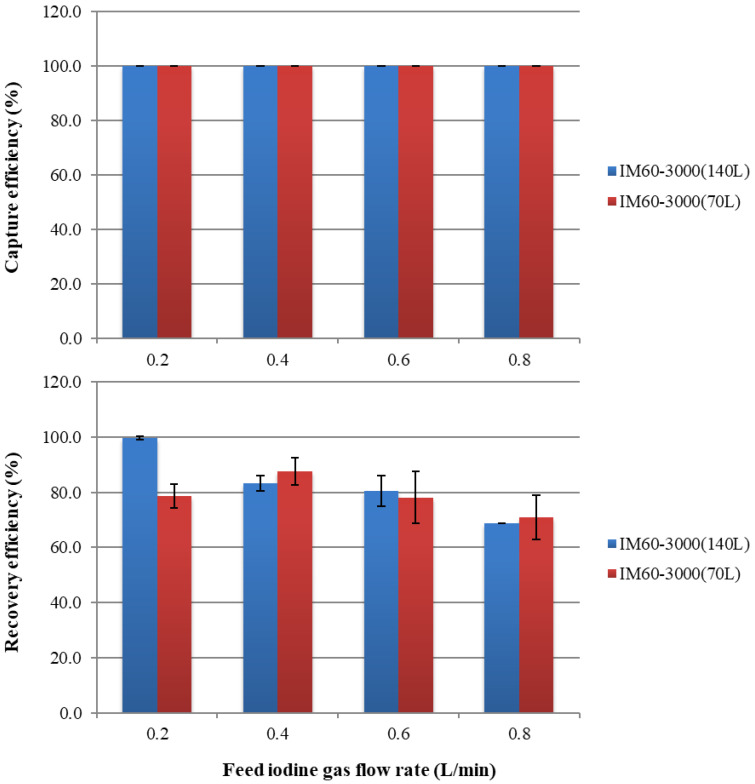
Capture efficiency and recovery efficiency of iodine in the gaseous phase using the improved SFMMs after 2 h. The upper and lower limits of the bars indicate the respective data, and the mean of the two points is plotted (*n* = 2).

Results obtained using the improved SFMMs are shown in Figure 10. Although both the capture and recovery efficiencies of conventional SFMMs decreased rapidly with increasing feed flow rates, both the capture and recovery efficiencies significantly improved with the improved SFMMs. Even with increasing gas flow rates, the iodine concentration at the outlet was almost zero, implying that the capture efficiency could be maintained at 100% at any flow rate. The recovery efficiency also increased significantly to ≥70% at all flow rates. Although the recovery efficiency is lower than the capture efficiency, iodine is 100% captured in the module; hence, recovery is possible by continuing to circulate the recovery solution. Unlike the conventional SFMM, the improved SFMM visually showed no uneven sorption, such as the one-sided flow depicted in Figure 8. The capture and recovery efficiencies indicate that a module length of 70 mm is sufficient and that the improved SFMM could efficiently separate and recover iodine in the gas phase.

Ion exchange resins, activated carbon, and zeolite are generally used to absorb and concentrate iodine [21,22,23,24]. For instance, liberated iodine is adsorbed onto an anion exchange resin. Iodine is eluted from the saturated resin by washing first with a strong alkaline solution and then with aqueous sodium chloride [25]. Hence, these processes are batch operations rather than continuous operations and require regeneration of the resin and activated carbon [26], leading to high costs. In contrast, iodine recovery using the PCD method with SFMMs is a continuous operation, and the silicone membrane does not require regeneration operations. Moreover, because silicone membranes are nonporous materials, they have low resistance to gas and liquid flow, contributing to lower operating costs.

Silicone is generally stable up to approximately −50 °C to 200 °C [27] and can be widely applied from liquid to gaseous phases. An iodine solution was flowed for 1 month through an SFMM with no damage to the hollow fiber [14]. When iodine gas was treated for 1 day at 0.8 L/min using the IM60-3000-70P module, approximately 1.2 m^3^ of gas could be processed. By increasing or decreasing the number of modules according to the processing volume, we demonstrated the potential to efficiently recover iodine at a low cost in a small space. 

## 4. Conclusions

We applied the PCD method for separating iodine in the gaseous phase using an SFMM, which could recover iodine in the gaseous phase. However, because of the reduction in the capture efficiency, due to the one-sided flow of iodine in the module, we improved the structure of the SFMM by placing a perforated pipe to guide the iodine gas to the center of the bundle of hollow fibers. The iodine gas flowed into the pipe, improving the structure of the SFMM, so that iodine could be efficiently diffused and adsorbed from the holes to the hollow fiber membranes arranged around the pipe. The improved module significantly increased both capture and recovery efficiencies. The long-term stability of performance is currently being investigated using this improved module. We propose this improvement as a highly effective structure for separating iodine and other components.

## Figures and Tables

**Table 1:** Specifications of silicone rubber hollow fiber membrane module (SFMM) used in this study.

Data	M60-3000 (70S)	M60-3000 (140S)	M60-3000 (70P)	M60-3000 (140P)	M100-1700 (70S)
Material of fiber	Silicone rubber				
Materials of housing	Polycarbonate				
Inner diameter of fiber (μm)	200	200	200	200	300
Membrane thickness (μm)	60	60	60	60	60
Length of fiber (mm)	70	140	70	140	70
Number of fibers	3000	3000	3000	3000	1700
Effective membrane area (m^2^)	0.17	0.34	0.17	0.34	0.15
Shape of fiber	Straight	Straight	Plain-fabric	Plain-fabric	Straight

**Table 2:** HPLC conditions for I_2_ measurement.

HPLC Model	LC-6A (Shimadzu Co., Kyoto, Japan)
Detector	UV spectrophotometric detector SPD-6A (Shimadzu Co.) 220 nm
Column	Luna 5 m C18, 150 mm × 4.6 mm (Phenomenex, CA, USA)
Column temperature	40 °C
Mobile phase	CH_3_CN/H_2_O-20 mM tetrabutylammonium phosphate = 25/75
	(Kanto Kagaku Co., Ltd., Tokyo, Japan)
Flow rate	1.0 mL/min

**Table 3:** Specifications of improved silicone rubber hollow fiber membrane module (SFMM).

Data	IM60-3000 (70P)	IM60-3000 (140P)
Material of fiber	Silicone rubber	
Materials of housing	Polycarbonate	
Inner diameter of fiber (μm)	200	200
Membrane thickness (μm)	60	60
Length of fiber (mm)	70	140
Number of fibers	3000	3000
Effective membrane area (m^2^)	0.17	0.34
Shape of fiber	Plain-fabric	Plain-fabric

## Data Availability

The original contributions presented in the study are included in the article; further inquiries can be directed to the corresponding author.

## Abbreviations

PCD	permeation and chemical desorption
SFMM	silicone hollow fiber membrane module
